# Impact of Heat Block Drying on Morphologic Preservation and Staining of Peripheral Blood Smears

**DOI:** 10.1111/ijlh.70200

**Published:** 2026-07-16

**Authors:** Ryan C. Shean, Nicolas Mavromatis, Gary Isom, Angie Turnbow, Lauren Pearson

**Affiliations:** ^1^ Department of Pathology University of Utah Salt Lake City Utah USA; ^2^ ARUP Laboratories Salt Lake City Utah USA

**Keywords:** drying, heat block, morphology, peripheral smear, standardization

## Abstract

**Introduction:**

Morphologic evaluation of the peripheral blood smear is critical for diagnosis and ruling out certain hematologic disorders. While air‐drying is standard, some laboratories may use heat blocks to expedite drying. Excessive heat may induce morphologic artifacts, but there is a lack of systematic literature on this practice.

**Methods:**

Thirteen residual clinical samples representing normal and pathologic conditions were prepared in triplicate and dried under three conditions: ambient air, 37°C, and 45°C heat blocks. Smears were stained and evaluated by two trained pathology residents using a 100‐cell WBC differential, semiquantitative grading of RBC forms (0–3+), and subjective smear quality scoring (0–5). Discrepancies were reconciled after unblinding.

**Results:**

Average smear quality was 3.5 (SD 1.1) with no significant difference across drying conditions (*p* > 0.05). WBC differential counts were unaffected by heat treatment. All diagnostically relevant morphologic findings (e.g., blasts, hypersegmented neutrophils, hereditary RBC forms) were identified in all conditions. However, smears dried at 45°C exhibited spurious morphologic changes, including increased schistocytes (5 cases), spherocytes (5 cases), and elliptocytes (3 cases).

**Conclusions:**

Heat block drying at 37°C yields morphology that approximates air drying but would require internal validation prior to implementation. In contrast, drying at 45°C possibly introduces clinically significant artifacts, risking false‐positive interpretations especially for hemolysis. Laboratories should avoid drying at high heat, internally validate any heat block use, and pursue standardized smear preparation protocols to minimize preanalytical variability.

## 1 Introduction

Morphologic evaluation of the peripheral blood is an important part of the diagnostic workup for many hematologic disorders. Peripheral blood smear findings can quickly and reliably raise suspicion for life‐threatening conditions including acute promyelocytic leukemia and thrombotic microangiopathies. Peripheral blood morphology may also be used to cheaply and quickly guide diagnosis and treatment of less acute conditions such as erythrocyte abnormalities, anemia, myeloproliferative and lymphoproliferative disorders, and myelodysplastic syndromes [1]. Although automated differentials are highly reliable for many cases, manual smear review remains important for suspected blasts, schistocytes, dysplasia, abnormal lymphoid cells, platelet clumping, and analyzer‐flag confirmation. Additionally, automatic analyzers are known to overcall certain morphologic findings, such as schistocytes, requiring confirmation before releasing results. Consensus guidelines from the International Council for Standardization in Hematology (ICSH) emphasize standardization of nomenclature and grading for peripheral blood cell morphology in order to improve reproducibility and communication of relevant findings [2]. Equally important as harmonizing nomenclature and grading parameters is standardization of slide preparation methods to obtain an optimal smear with minimal artifacts and therefore an accurate morphologic assessment.

There are many causes of artifactual findings on peripheral blood smears. Despite widespread informal knowledge of many of the common artifacts, there is limited literature with systematic studies describing artifactual findings and their causes. One of the most common and well‐described morphologic artifacts is pseudothrombocytopenia, or artifactual platelet clumping, typically caused by EDTA‐dependent antibodies already present in the patient [3].

Storage artifacts are relatively common in EDTA collected blood and begin to develop in under 4 h [4]. Morphologic changes related to prolonged storage of EDTA collected blood include cytoplasmic vacuolation of nucleated cells, platelet aggregation, crenation of erythrocytes and increased spherocytes [4]. Additionally, metabolically active cells such as neutrophils may develop changes resembling toxic changes after prolonged storage [5]. Prolonged storage has also been described to cause artifactual loss of Rouleaux formation in pathologic samples [6].

After collection, transport, and possible storage, the blood film must be prepared. High humidity during slide preparation has been associated with artifactually increased numbers of stomatocytes and target cells [2]. Although present in the rare condition of hereditary stomatocytosis, stomatocytes are almost always an artifact. In an unpublished study, Robert V. Pierre found that 42% of blood films had significant numbers of stomatocytes [6]. Additionally, wedge preparation methods can differentially push larger cells such as monocytes or blasts to the feathered edge of the smear, and may lead to 5%–10% underestimation of monocyte count when compared to flow cytometry differential counts [7]. Blood exposure to glass slides can lead to artifactual echinocyte formation, likely due to pH increase as alkali releases from the glass [6]. Echinocytes may also result from flattening erythrocytes on the glass slide [8]. If the smear is too thick, artifactual Rouleaux formations may be seen.

After preparation, the slide must be dried. In Western countries, most smears are naturally air‐dried. Manufacturer instructions for automated instruments and procedural references for manual techniques frequently stress that thorough air‐drying is necessary to avoid water artifacts and staining variability that can significantly alter blood film morphology [9]. If a slide is dried too slowly, torocytes (donut cells) may be artifactually increased [8]. Several Japanese researchers have described the practice of air‐drying smears with a fan, describing better resolution for appreciating fine intracellular structures [10]. It is thought that the natural air‐drying method may cause cells to shrink, particularly when there is high humidity [11]. In veterinary pathology, using a heat block at a low setting or hair blow dryer is described as a method of minimizing refractile markings that can distort erythrocyte morphology [12].

However, excessive heat has been shown to fragment red blood cells both in vivo and ex vivo [13, 14] which may lead to artifactually increased schistocytes and spurious suspicion for microangiopathic hemolytic anemia. Thermal injury studies on whole blood and erythrocytes demonstrated extensive fragmentation and agglutination after in vitro heating, leading to critical changes in hematologic parameters [14]. Additionally, after in vitro heating, peripheral smear morphology was significantly altered, with increased spherocytes, echinocytes, and fragments noted [15]. These artifactual findings were typically noted to occur above 45°C–50°C. It is biologically plausible these findings extend to drying peripheral blood smears with heat blocks at excessive temperatures.

Although College of American Pathology (CAP) accreditation checklists and Clinical and Laboratory Standards Institute (CLSI) standards emphasize control of preanalytical variables and quality standards for manual blood film examination, they do not explicitly specify drying temperatures or methodology, leaving room for practice variation [16, 17]. Upon review of the literature, we found no focused studies systematically testing the use of heat blocks during thin blood smear preparation; most prior work focuses on thermal treatment of stored blood or general storage and drying artifacts. Additionally, prior published work has focused primarily on normal erythrocyte morphology and the effects of heat blocks on pathologic samples as well as white blood cell (WBC) morphology, which is not well described.

Due to a satellite lab at our institution implementing a heat block drying step in an attempt to reduce turnaround times, we embarked on the following qualitative QI project to evaluate how different heat block temperatures affect the quality and interpretation of peripheral blood smears intended for manual pathologist review. Based on these findings, we aim to propose best‐practice temperature limits and workflow recommendations that preserve smear morphology, while aligning with ICSH, CAP, and CLSI standards for standardization and reproducibility.

## 2 Materials and Methods

Residual samples from routine clinical work were collected. Cases arriving for routine clinical work were manually selected after final sign out. Out of the diagnoses encountered during the study period (approximately 1 week), cases were retrospectively reviewed and manually selected to cover a variety of pathological conditions (Table 1). These conditions were not pre‐specified and included anemia, chronic myelomonocytic leukemia, chronic lymphocytic leukemia, normal smears, and others. In all cases, the clinical diagnosis was assigned from retrospective chart review based on appropriate diagnostic laboratory testing (bone marrow integrated pathology reports, iron/B12 labs, etc.) and clinical history.

**TABLE 1:** Clinical diagnoses and semiquantitative summary of relevant morphologic interpretation is provided.

Case #	Diagnosis		Ambient			37C			45C		Quality
		RBC	WBC	PLT	RBC	WBC	PLT	RBC	WBC	PLT	
1	IDA	Anisopoikilocytosis, cigar cells	Normal	Normal	Anisopoikilocytosis, cigar cells	Normal	Normal	Increased anisopoikilocytosis, cigar cells, 3+ schistocytes	Rare smudge cells, occasional blasts	Normal	2.8
2	Normal	Normal	Normal	Normal	1+ elliptocytes	Normal	Normal	2+ elliptocytes	Distorted granulocytes	Normal	3.8
3	Liver disease	Target cells and acanthocytes	Normal	Normal	Target cells and acanthocytes, 1+ spherocytes	Normal	Normal	Target cells, acanthocytes, 3+ spherocytes	Degenerating/distorted granulocytes	Normal	3.8
4	CMML	Normal	Atypical immature monocytes	Normal	Normal	Atypical immature monocytes	Normal	Moderate anisopoikilocytosis, 2+ spherocytes	Atypical immature monocytes	Normal	4.3
5	CML	Normal	Left shift increased basophils	Normal	Normal	Left shift increased basophils	Normal	Normal	Left shift increased basophils	Normal	2.3
6	B12 deficiency	Normal	Hypersegmented neutrophils	Normal	2+ schistocytes	Hypersegmented neutrophils	Normal	Moderate anisopoikilocytosis,1+ elliptocytes, 1+ schistocytes	Hypersegmented neutrophils, rare blasts	Normal	4.2
7	CLL	Normal	CLL‐like	Normal	1+ elliptocytes	CLL‐like	Normal	1+ schistocytes, 2+ elliptocytes	CLL‐like, degenerating neutrophils	Normal	3.7
8	Hereditary elliptocytosis	3+ elliptocytes	Normal	Normal	3+ elliptocytes	Normal	Normal	3+ elliptocytes, 3+ spherocytes	Normal	Normal	3.7
9	MDS	Moderate polychromasia and anisopoikilocytosis	Very atypical granulocytes	Normal	Moderate polychromasia and anisopoikilocytosis	Very atypical granulocytes	Normal	Moderate polychromasia and anisopoikilocytosis	Very atypical granulocytes, increased vacuolation and degenerating neutrophils	Normal	2.3
10	HS	Moderate anisopoikilocytosis	Normal	Normal	Moderate anisopoikilocytosis, 1+ echinocytes	Normal	Normal	Moderate anisopoikilocytosis, 1+ schistocytes	Lymphocytes with cytoplasmic blebbing	Normal	3
11	T‐PLL	Mild anisopoikilocytosis	Left shift and toxic granulation, markedly increased prolymphocytes	Normal	Mild anisopoikilocytosis	Left shift and toxic granulation, markedly increased prolymphocytes	Normal	Mild anisopoikilocytosis, 1+ spherocytes	Left shift and toxic granulation, markedly increased prolymphocytes	Normal	3.7
12	IDA	Moderate anisopoikilocytosis	Normal	Normal	Moderate anisopoikilocytosis, 1+ schistocytes	Normal	Normal	Moderate anisopoikilocytosis, 1+ spherocytes, 2+ schistocytes	Normal	Normal	4.8

Abbreviations: CLL, chronic lymphocytic leukemia; CML, chronic myeloid leukemia; CMML, chronic myelomonocytic leukemia; HS, hereditary spherocytosis; IDA, iron deficiency anemia; MDS, myelodysplastic syndrome; T‐PLL, T‐cell prolymphocytic leukemia.

Only samples with sufficient volume for making a standardized smear in triplicate were included and all samples were identified and prepared within the laboratory's routine turn‐around time (< 24 h between specimen receipt and smear preparation). Because EDTA‐related storage artifacts may occur within 4 h of collection [18, 19] each specimen was compared only with the alternate drying conditions for each case.

### 2.1 Smear Preparation and Staining

Peripheral blood was collected in EDTA and prepared by trained hematology laboratory staff credentialed to prepare clinical specimens. All smears were prepared in triplicate. Briefly, smears were created with the manual wedge push preparation method and then dried prior to staining. Drying was performed in each of the following conditions: air drying following lab standard operating procedure for 600 s, placed on a heat block at 37+/−2°C for 600 s, and placed on a heat block at 45 (+/−) 2°C for 600 s. Heat block temperature was controlled and standardized with NIST calibrated thermometers. As peripheral smear preparation is susceptible to variation in temperature and humidity, all procedures were performed in a temperature‐ and humidity‐controlled laboratory. The acceptable temperature and humidity range in the lab was 20°C–25°C and 15%–55%, respectively. However, during the study period the observed ambient temperature fluctuated between 21.2°C–23.3°C and the observed humidity between 27.9%–38%. After drying, smears were Wright‐Giemsa stained according to the lab's standard operating procedures.

Samples were blinded and randomized. Morphologic evaluation was performed by two pathology residents with dedicated training in morphologic evaluation of the peripheral blood and qualified for clinical responsibility manually reviewing peripheral smear interpretation requests during routine clinical work at the hospital. Slides were assessed with a standard 100 cell WBC differential count. Erythrocyte morphology was assessed with semiquantitative grading (0–3+) per ICSH guidelines [2] for echinocytes, schistocytes, elliptocytes, acanthocytes, target cells, spherocytes, dacrocytes, and nucleated erythrocytes. Additionally, subjective comments were collected for RBC, WBC, and platelet morphology, as well as free text comments for the entire smear. Finally, a semiquantitative subjective score for smear quality was assessed for each slide. The scale used was as follows: 0—uninterpretable, 1—major issues and artifacts that may impact the final interpretation, 3—minor issues/artifacts that appear unlikely to impact final interpretation, 4—rare or very minor issues/artifacts with no impact for final interpretation, and 5—no identifiable issues or artifacts. After independent scoring, the slides were unblinded to annotate and photograph any perceived heat‐related artifacts and reveal and reconcile any major discrepancies.

### 2.2 Ethics

Use of de‐identified residual specimens was performed under institutional quality improvement protocol and IRB#00077285 as waived research. No additional phlebotomy was required and informed consent was not required.

### 2.3 Statistical Analysis

Statistical analysis was performed in R v4.4.1 [20]. No formal sample‐size calculation was performed because this was a semi‐qualitative QI project using residual specimens. Statistical tests included chi‐squared analysis to assess overall comparison in semi‐quantitative morphology findings between conditions, Fisher's exact test to assess differences in individual morphology findings, Cohen's linear weighted kappa to assess agreement between observers, and two sample *t*‐tests to assess differences between smear quality.

## 3 Results

A total of 13 unique patient samples across a range of pathological and normal conditions, with relevant morphologic considerations were collected (Table 1). All slides were dried in each condition for 600 s. The average subjective smear quality across all slides and cases was 3.5 (SD 1.1, range 2–5). Distribution of smear quality did not differ significantly by heat treatment category (two sample *t*‐test; *p* > 0.05). No statistically significant differences in 100 cell differential count (neutrophil, lymphocyte, monocyte, or blast count) were noted between heating conditions for all cases (*p* > 0.05). In cases with diagnostically important morphologic findings (hypersegmented neutrophils, blasts, CLL lymphocytes, MDS, immature monocytes, hereditary elliptocytosis, and hereditary spherocytosis) the relevant findings were noted in all heating conditions. Agreement between overall semiquantitative RBC morphology grades was assessed by chi‐squared test with no significant differences (*p* > 0.05) and by Fisher's exact test on each type of abnormal erythrocyte form (echinocytes, schistocytes, elliptocytes, acanthocytes, target cells, spherocytes, dacrocytes and nucleated erythrocytes) and revealed no significant differences (*p* > 0.05). Agreement for differential counts between graders was high (Cohen's linear weighted kappa: 0.8671, mean absolute difference 3.2%). However, two cases that were clinically negative for blasts and did not have blasts noted at room temperature or 37°C had blasts identified only at 45°C (1%–4%).

Between all cases, there was no difference between heat treatment conditions for dacrocytes or target cells. There were five cases that had increased spherocytes only noted in slides exposed to heated conditions (37°C or 45°C on heat blocks). There were also five cases that had schistocytes identified only in heated conditions (Figure 1A,B). There were no cases where schistocytes were identified only after air‐drying. There were also three cases with elliptocytosis identified only in heated conditions. There was no difference between heat treatments for platelet clumping. Additionally, there were no differences noted for platelet morphology, size, and granulation between drying conditions.

**FIGURE 1:**
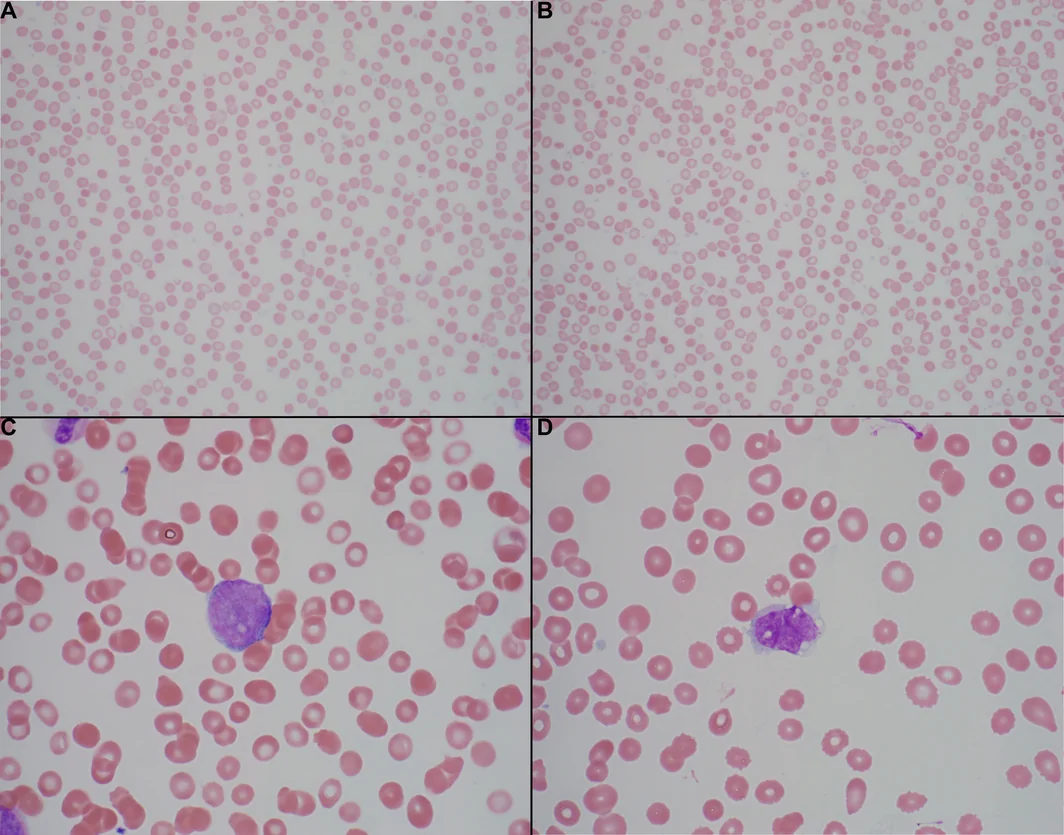
Effect of heat blocks on peripheral smear morphology. Erythrocytes from the same patient are shown air‐dried (A) and dried at 45°C (B). Increased fragmentation, crenation, and distortion of morphology are noted in the heated condition as compared to the air‐dried sample. An illustrative example of leukocyte morphology changes is shown for the same sample air dried (C) and heated at 45°C (D). Relevant changes noted include nuclear and cytoplasmic distortion and increased vacuolation in the heated sample.

Upon review of subjective comments, slides dried on heat blocks were noted to have more distorted leukocyte morphology. After unblinding, this was manually examined and leukocytes exposed to the heat block at 45°C had more irregular cytoplasmic and nuclear borders, slightly decreased size, and increased cytoplasmic vacuolation (Figure 1B,C). Additionally, after unblinding, the cases with artifactually increased schistocytes, spherocytes, and elliptocytes only in heated conditions were manually reviewed and confirmed.

## 4 Discussion

We present an assessment of smear quality using heat blocks at 37°C and 45°C compared to “standard” air drying. To our knowledge this is the first study to specifically study the effect of heat blocks on smear preparation, as well as examine potential effects on pathologic specimens and WBC morphology. Overall, we found that heat block drying at 37°C produced smear morphology largely indistinguishable from ambient drying, and differences were generally within expected intra‐observer variation for manual review of peripheral blood smears [17, 21, 22]. Despite this, smears dried at 37°C on a heat block occasionally showed increased spherocytes and elliptocytes relative to the air‐dried smears. As a result, exposure of glass slides to even “physiologic” temperatures may still distort morphology and any lab considering drying at this temperature would need to clinically validate this process to ensure any potentially introduced artifacts are acceptable.

Additionally, smears dried at 45°C occasionally demonstrated morphology that could be clinically different from smears dried at 37°C or air dried. Clinically relevant spurious findings potentially introduced because of heat treatment included erythrocyte abnormalities, such as increased identification of elliptocytes, spherocytes, and most concerningly, schistocytes. Spurious erythrocyte fragmentation raises the risk of increased false positive impressions for processes such as microangiopathic hemolysis, uremia‐related burr cells, or thermal injury, potentially triggering unnecessary workups. These findings align with prior thermal injury studies on stored blood showing temperature dependent RBC membrane alterations and increased fragmentation at or above 45°C and other studies examining storage artifacts of peripheral blood. Additionally, the distorted leukocyte morphology noted more often in the 45°C condition (irregular cytoplasmic and nuclear borders, slightly decreased size, and increased cytoplasmic vacuolation) could lead to possible misclassification of cell type, potentially into alarming categories such as blasts, and could possibly lead to spurious requests for pathology review of abnormal/atypical cells. Although potentially artifactual findings, especially 1%–4% blasts, could be explained by random variation in 100 cell differentials, this work provides support for the possibility of clinically significant spurious findings in heat treated peripheral smears.

Interestingly, subjective quality scores were very similar across cases and did not vary much by heat condition. Possible explanations for this may be that time between collection and smear preparation varied between 1 and 24 h in our study and that quality scores overall were generally very high (average 3.5 in all conditions). Another potential explanation for this is that the factors used to determine overall subjective smear quality (stain quality, thickness of smear, size of evaluable area, etc.) are not affected by heat treatment.

A major limitation of this study is the small sample size, which limits the power and utility of statistical tests, as well as the overall generalizability of these findings. However, these findings are biologically plausible and consistent with previously published literature on the effect of thermal injury on mammalian blood.

Additional limitations include a single center design, lack of a prospective design, and subjective grading. A key limitation of this study is the interval between specimen collection, receipt, and smear preparation was variable. Although all slides were prepared according to our routine clinical laboratory practice, which allows < 24 h from receipt to preparations, EDTA can introduce artifacts after 4 h. This likely contributed to much of the case‐to‐case variation in subjective smear quality. However, labs with outlying clinics may have difficulty preparing smears in under 4 h, and our findings may be generalizable in these settings. Furthermore, as all slides were prepared manually, findings may not generalize to automated slide stainers or other stains. An additional limitation is that due to the small number of total samples and reliance on residual specimens, several important diagnoses with relevant peripheral smear findings such as thalassemia, DIC, TTP, renal failure, and others were not included in our study set.

Future work might increase the total number of samples, employ a prospective design, specifically investigate humidity, timed exposure, explore additional temperatures, or utilize an automatic slide stainer.

As laboratories and staff are frequently under pressure to report CBC with differential results expediently, there may be increased interest in implementing potentially time‐saving measures. Indeed, this project is a result of a local satellite lab experiencing external pressure to release differential counts requiring slide preparation and implementing heat blocks to meet the clinical demand. In our case, the lab had a preconceived perception that heat blocks would save time. However, before the practice was stopped, it appeared that there was minimal or no time saving in smear preparation.

These pressures may lead other labs to also consider perceived time saving steps such as heat blocks, however, given the potential deleterious impacts, any proposed time‐saving change must be validated. We did not validate or use heat blocks for this purpose. However, a reasonable validation in this case could consist of measuring and quantifying the variation relative to the set temperature over time, determining the acceptable temperature range, and then determining the overall impact on quality with a study designed to reach statistical significance to show at least non‐inferiority.

Based on our project it appears that because of the clinically significant morphological abnormalities that could possibly appear when preparing slides with heat blocks at excessive temperatures, laboratories should avoid drying at high heat (> 37°C). If laboratories are considering use of heat blocks at any temperature, validation must be performed to determine fluctuation and variability in temperature output and smear quality. In our experience, operationally deployed heat blocks are difficult to maintain at 37°C with appropriate temperature variance. Labs considering this process must determine an acceptable range that does not negatively impact morphology. Furthermore, standardization in smear preparation techniques could be pursued by ICSH and/or CAP to ensure that rigorous adherence to standardized reporting criteria and language is not compromised by allowable variations in slide preparation techniques.

## Author Contributions

All authors designed the study, analyzed data, and wrote the final manuscript.

## Funding

The authors have nothing to report.

## Consent

Use of de‐identified residual specimens was performed under institutional quality improvement protocol. No additional phlebotomy was required and informed consent was not required.

## Conflicts of Interest

Ryan C. Shean, Nicolas Mavromatis, Gary Isom, Angie Turnbow: Declare no relevant conflicts of interest. Lauren Pearson: Consulting for Beckman Coulter.

## Data Availability

The data that support the findings of this study are available from the corresponding author upon reasonable request.
